# Differential Treatments Based on Drug-induced Gene Expression Signatures and Longitudinal Systemic Lupus Erythematosus Stratification

**DOI:** 10.1038/s41598-019-51616-9

**Published:** 2019-10-29

**Authors:** Daniel Toro-Domínguez, Raúl Lopez-Domínguez, Adrián García Moreno, Juan A. Villatoro-García, Jordi Martorell-Marugán, Daniel Goldman, Michelle Petri, Daniel Wojdyla, Bernardo A. Pons-Estel, David Isenberg, Gabriela Morales-Montes de Oca, María Isabel Trejo-Zambrano, Benjamín García González, Florencia Rosetti, Diana Gómez-Martín, Juanita Romero-Díaz, Pedro Carmona-Sáez, Marta E. Alarcón-Riquelme

**Affiliations:** 1 Centro de Genómica e Investigaciones Oncológicas Pfizer-Universidad de Granada-Junta de Andalucía (GENYO), Granada, Spain; 2 Johns Hopkins University School of Medicine, Baltimore, Maryland USA; 3 GLADEL consultant, Rosario, Argentina; 4 Centro Regional de Enfermedades Autoinmunes y Reumáticas, Rosario, Argentina; 5 Centre for Rheumatology, Division of Medicine University College London, London, United Kingdom; 6 Department of Immunology and Rheumatology, Instituto Nacional de Ciencias Médicas y Nutrición “Salvador Zubirán”, Mexico City, Mexico; 7 Unit of Chronic Inflammation, Institute for Environmental Medicine, Karolinska Institutet, Stockholm, Sweden

**Keywords:** Systemic lupus erythematosus, Machine learning, Functional clustering, Statistical methods

## Abstract

Systemic lupus erythematosus (SLE) is a heterogeneous disease with unpredictable patterns of activity. Patients with similar activity levels may have different prognosis and molecular abnormalities. In this study, we aimed to measure the main differences in drug-induced gene expression signatures across SLE patients and to evaluate the potential for clinical data to build a machine learning classifier able to predict the SLE subset for individual patients. SLE transcriptomic data from two cohorts were compared with drug-induced gene signatures from the CLUE database to compute a connectivity score that reflects the capability of a drug to revert the patient signatures. Patient stratification based on drug connectivity scores revealed robust clusters of SLE patients identical to the clusters previously obtained through longitudinal gene expression data, implying that differential treatment depends on the cluster to which patients belongs. The best drug candidates found, mTOR inhibitors or those reducing oxidative stress, showed stronger cluster specificity. We report that drug patterns for reverting disease gene expression follow the cell-specificity of the disease clusters. We used 2 cohorts to train and test a logistic regression model that we employed to classify patients from 3 independent cohorts into the SLE subsets and provide a clinically useful model to predict subset assignment and drug efficacy.

## Introduction

Systemic lupus erythematosus (SLE) is a heterogeneous autoimmune disease characterized by unpredictable patterns of flares and remission. During periods of active disease a wide range of clinical symptoms may appear, ranging from cutaneous involvement to severe organ damage^1^. Disease activity can be measured using a global score such as the SLE Disease Activity Index (SLEDAI)^2^, and variations therein, such as the SLEDAI-2K or SELENA-SLEDAI. Patients characterized with similar disease activity scores may have different prognoses and clinical manifestations^3^.

Previously, we reported a longitudinal stratification of SLE patients based on clustering analysis of a expression data and SLEDAI correlation matrix^4^. Analyzing two independent longitudinal cohorts, we found a robust stratification of SLE patients into three main groups characterized by differences in the correlated genes that reflected how immune cell populations evolved with disease activity. The three subtypes showed specific associations with particular clinical parameters. The increase in disease activity in two of the groups was linked to an increase in the percentage of neutrophils, while in the third group disease activity increases were related to lymphocyte percentage increases. In addition, the neutrophil-related groups had a higher risk of developing proliferative nephritis, they were associated to lower C3 and C4 protein levels and lymphopenia, and molecular functions related to IFN I, apoptosis-related pathways, and cytokine signaling pathways were over-represented. The lymphocyte-related group had a higher incidence of Sjögren’s syndrome, an elevated incidence of abnormal liver function, and the main altered pathways were related to lymphocyte T and B signaling and activation.

The heterogeneity of SLE is also reflected in the variability in drug responsiveness between different patients, which results in a large proportion of patients showing no or partial response to therapies and where this trial-and-error approach clearly requires improvement^5^.

Previous studies conducted in other contexts, such as cancer, have shown that the cellular percentage, measured as the neutrophil to lymphocyte ratio (NLR)^6^ could play a fundamental role in drug responsiveness^7,8^. However, the relationship between cell types and drug efficacy or even drug selection has not been studied in autoimmune diseases. Therefore, we propose that the longitudinal classification associated with the fluctuation of neutrophils and lymphocytes could be used to study the plausibility of a better drug selection specifically within each group potentially to improve the treatment efficacy and eventually reduce costs.

*In-silico* drug-repurposing analysis based on gene expression data allow the identification of new therapeutic indications for a condition based on drugs used in other contexts by measuring the theoretical capability of a drug to revert a pathological gene expression signature. The gene expression signature of a disease is compared against a large collection of profiles derived from different compounds, obtaining a similarity or connectivity score. A negative similarity score suggests that the effect of the drug on gene expression is opposite to the effect of the disease, and it is reasonable to hypothesize that the drug might be able to revert the disease gene expression program and potentially the phenotype itself. A positive similarity score suggests that the compound produces a similar gene expression pattern to that of the disease^9,10^. CLUE (https://clue.io) is a cloud-based platform for the analysis of a large panel of perturbation-driven gene expression data from more than 8,000 compunds^11^, some of them commonly used in autoimmune diseases. Drug-repurposing analysis have been previously performed in SLE^12,13^, But these studies did not seek differential responses of treatments based on SLE heterogeneity, a strategy that has been used in cancer^14^. Finally, if indeed clustering signifies a difference that may guide therapy selection, a classification model would be required for the physician to be able to assign a patient to the appropriate cluster or group of patients. Most importantly, such classifiers should be easy to use with routine laboratory and clinical information.

The principal aim of this work was to investigate if the subtypes of SLE previously described also showed differences in their association to drug-induced gene expression signatures analyzing the similarity scores among drug and subtype-based signatures. The second aim was to build a supervised classifier able to predict SLE subtype for individual patients using routine clinical laboratory information. The model provides a data-driven approach that migh help clinicians for subgroup SLE patients.

## Results

### Data cohort

For transcriptomic profile-based analysis we selected the visit with the higher SLEDAI for each patient shared with the longitudinal stratification study^4^. Cohort1 retained 16440 genes from 27 SLE adult patients with SLEDAI >5 and 20 healthy controls. Cohort2 was composed of a total of 15682 genes from 67 SLE pediatric patients and 72 healthy controls. For clinical based analysis, we used all patients with at least 3 visits with full clinical information, leading to a sample size of 38, 74, 192, 121, and 30 patients for cohorts 1, 2, 3, 4 and 5, respectively (Supplementary Dataset, Sheet 1).

### Clustering analysis of SLE patients based on drug signatures

**Figure 1:**
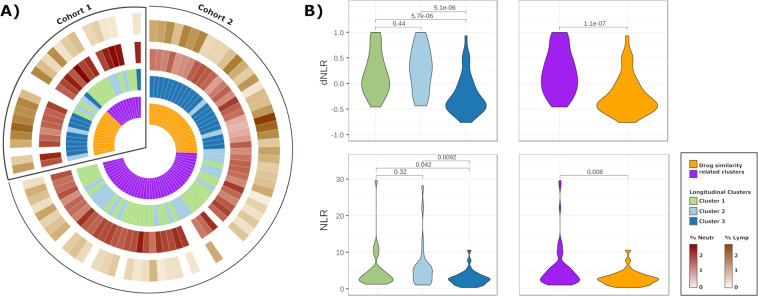
Clustering based on drug connectivity scores. (**A**) The assignment of each patient to the clusters based on the drug connectivity scores (violet and golden clusters), the assignment of each patient to each of the 3 longitudinal clusters, and the percentage of neutrophils and lymphocytes for each patient. (**B**) Summary of the comparison done with ANOVA of NLR and of the correlation between NLR and SLEDAI score values of patients from the different SLE subgroups. NLR: Neutrophil to Lymphocyte ratio. dNLR: correlation between NLR and SLEDAI across diferent time points.

Consensus clustering analysis of the drug by sample connectivity score matrix yielded two clear groups in both datasets (Fig. Supplementary 1). Figure 1A shows the membership of each patient in the clusters based on the drug connectivity scores, to the longitudinal clusters previously described^4^, and the percentages of lymphocytes and neutrophils for each patient. We observed in both cohorts that patients belonging to longitudinal clusters 1 and 2 (or neutrophil-related clusters) were grouped together in a single group based on the drug scores, while patients from cluster 3 (related to lymphocytes), were grouped separately. This suggests that there might be differences in the efficacy of disease signature reversion by drugs depending on whether the patient belongs to the longitudinal clusters 1 and 2, or whether the patient belongs to cluster 3. Figure 1B shows how both the longitudinal correlation between the NLR and the SLEDAI, as the NLR measured in a time point of active SLE, is significantly higher in the neutrophil-driven groups, which suggests the possible use of the cell ratio when taking therapeutic decisions.

### Drug connectivities in the SLE subgroups

**Figure 2:**
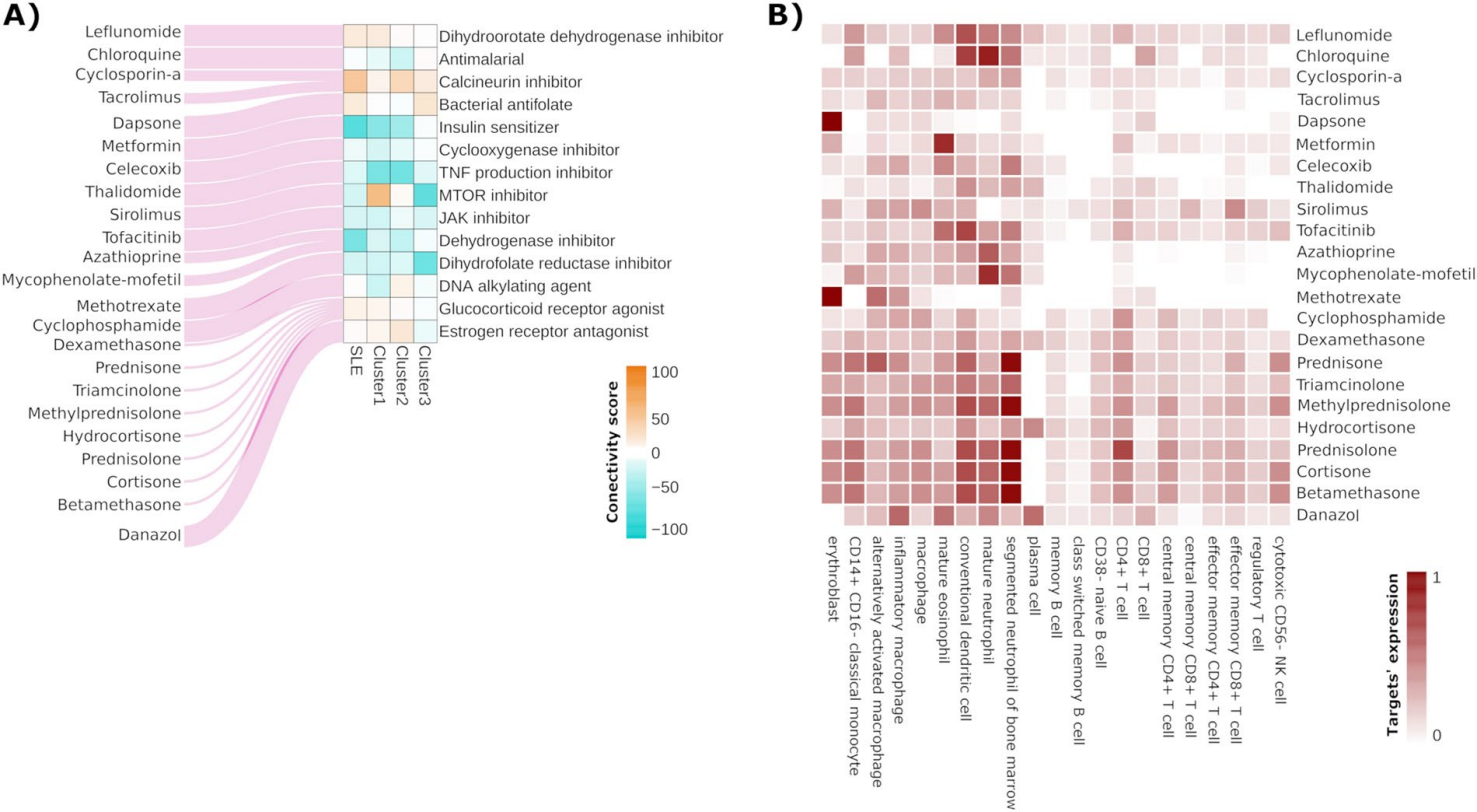
Analysis of drugs commonly used in SLE. (**A**) The connectivity scores of the MOAs on which the drugs commonly used in SLE act, shown for each subgroup and for a gene signature derived from all the patients together. On the left, the drugs are shown for each MOA. (**B**) The expression of the targets of each drug in the different cell types. The values are obtained by multiplying a binary matrix that contains the information of the targets for each drug and a matrix that contains the expression of each target in the different cell types.

A meticulous bibliographic search of drugs used in SLE yielded a list of 23 drugs also contained in CLUE. Figure 2A shows the connectivity score for MOAs from the drugs commonly used in SLE. We observed strong differences between the neutrophil and the lymphocyte-driven subgroups and also in respect to an “all SLE-patient”-derived signature. This allowed us to hypothesize that these drugs could differ in their efficacy depending on the cluster to which the patient belonged. The strongest differences were observed for mTOR inhibitors, which appeared to be able to revert the signature of the lymphocyte-driven subgroup best, and the TNF inhibitors in the case of the neutrophil-driven subgroups. The rest of the drugs did not reach very negative connectivity scores. Figure 2B shows the relative expression in blood cell types of the targets for each drug. Interestingly, targets for Sirolimus, an inhibitor of the mTOR signaling pathway, and for Thalidomine, that can act as a TNF inhibitor, were mostly expressed on lymphocytes and neutrophils, respectively.

**Figure 3:**
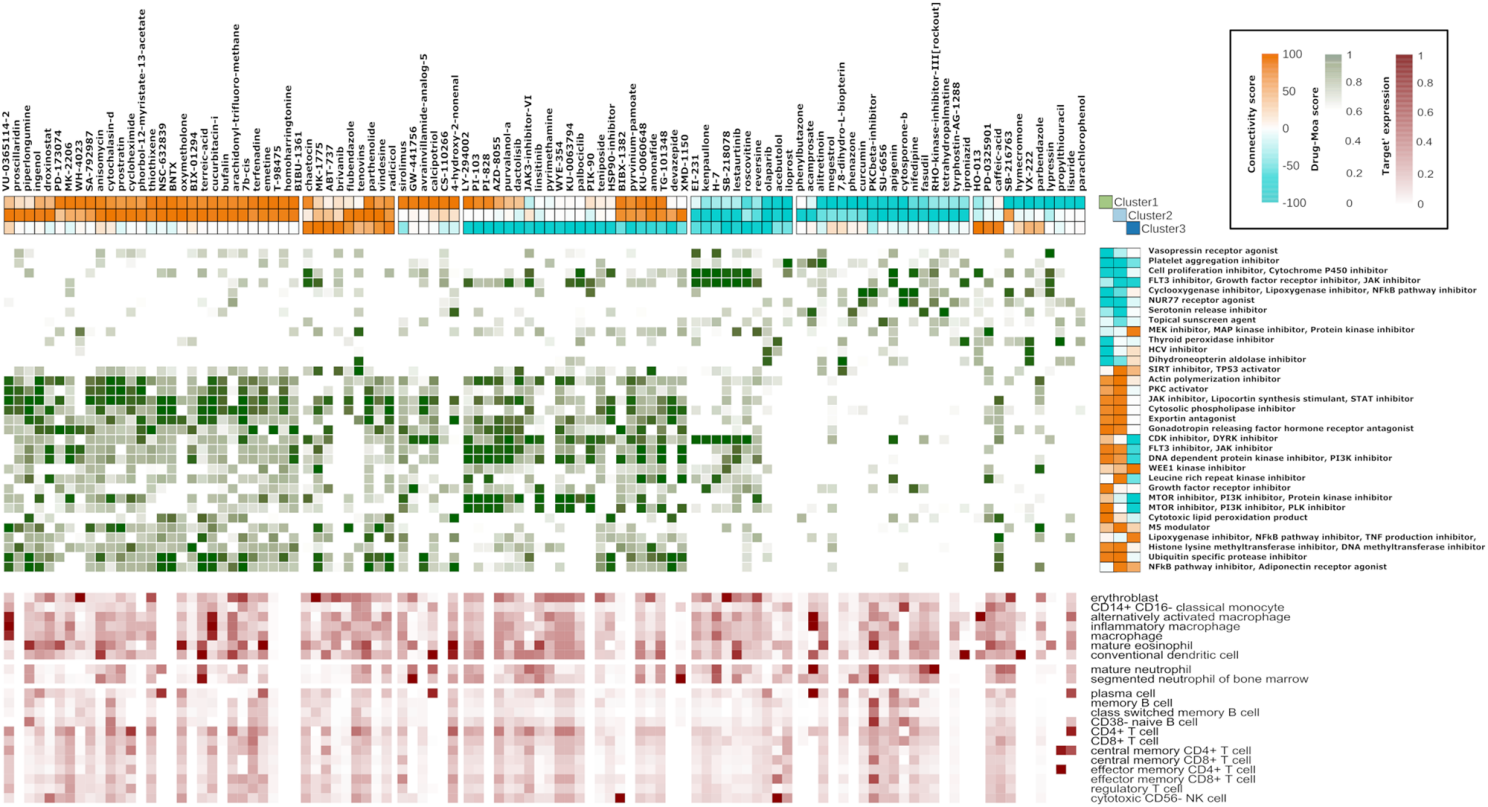
Drug-repurposing on SLE subgroups. Top) Significant drugs (score > = |90|) for at least one cluster. Right) Significant MOAs obtained using matrix multiplication. The green color represents the similarity level between MOAs and drugs obtained by GSEA. Bottom) Red color intensity represents the expression of targets for each drug in the different blood cell types.

The drug repurposing analysis yielded 104 drugs and 33 MOAs with significant scores (>|90|) in at least one of the clusters. Figure 3 shows the similarity scores between each cluster and the significant drugs (top), as well as the MOAs (right). As we previously observed, neutrophil-driven clusters shared strong similarities (Cluster 1 and 2) in comparison with the lymphocyte-driven cluster (Cluster 3). We observed different patterns of drug responses according to the connectivity score between clusters: drugs that induced similar gene profiles to drugs with signature reversion capability for the 3 clusters. Green color intensity represents the relationship based on the GSEA score between drugs and MOAs. Our results suggest that drugs related with inhibition of the mTOR and PI3K signaling pathways, and linked to anti-apoptotic and cell proliferation processes^15^, are the best candidates for Cluster 3, or lymphocyte-driven SLE. CDK inhibitors consistently may reverse the signature of the 3 clusters, and drugs aimed at regulating other apoptotic pathways, such as cyclooxygenase, JAK or NFkB inhibitors, could work best in neutrophil-driven SLE^16,17^. At the bottom, the figure shows the target expression of each drug in the different blood cell types.

The gene signatures clearly reflected the altered biological mechanisms. A functional analysis of these (Supplementary Fig. 2 and Dataset, sheets 2–4) revealed processes related to neutrophil-mediated activation and defense as the most significantly altered pathways in the neutrophil-driven subgroups, as well as autophagy and processes derived from cell stress, while cell proliferation and cell cycle were especially over-expressed in Cluster 3. The significant drugs obtained were directed to these pathways, as the inhibitors of mTOR for Cluster 3 or the inhibitors of rho kinase for the neutrophil-driven clusters, which regulate autophagy^18^.

### Classification model based on clinical data

The values of correlation between neutrophils and SLEDAI across different time points resulted in the best parameter to classify patients, reaching the highest accuracy (Supplementary Fig. 3). Therefore, the classification model *f*(*x*) obtained from K-fold cross validation is as follows:

$$f(x)=0.3438+1.9848\ast x$$

$$f(x)\{\begin{array}{ccc}Neutrophil\,driven\,SLE & if & f(x) > 0.18\\ Lymphocyte\,driven\,SLE & if & f(x)\le 0.18\end{array}$$

Where *x* is the correlation between neutrophil percentage and SLEDAI for a patient taking into account at least 3 time points.

**Figure 4:**
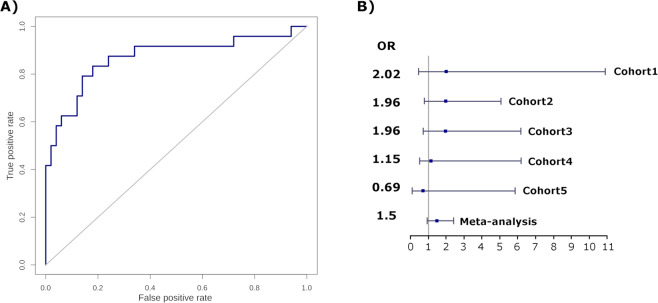
Classification model specificity and sensitivity. (**A**) ROC curve obtained when testing the classification model on the test set. (**B**) Odds-ratios (OR) and confidence intervals at 95% of suffering from nephritis when assigned to the neutrophil or to the lymphocyte-driven subgroup in each study, and in the meta-analysis.

Figure 4A shows a ROC curve obtained from the test set (Cohort2) in which the area under the curve (AUC) of 0.87 was obtained with a classification success of 80% of the patients assigned into the neutrophil or the lymphocyte-driven subgroups. Figure 4B shows the results of the meta-analysis of severe nephritis in patients from the 5 cohorts classified according to our model, in which we obtained a combined probability of 1.5 times higher risk with a tendency towards significance (p value = 0.09) of manifesting nephritis in the neutrophil-driven subgroup with respect to the lymphocyte-driven subgroup. In this type of meta-analysis, the p value is influenced both by the sample size and by the variance of the data. The incidence of nephritis is not exclusive to a group, although it is greater in one group than in another, for which the p value does not become significant for the threshold of 0.05. Only cohort 5 showed an OR in a different direction from the other cohorts, which could be due to its small sample size. The p value obtained by leaving out this cohort is 0.06.

## Discussion

In this exploratory analysis, we reported that previously established SLE subgroups^4^, notably, the neutrophil and lymphocyte-driven subgroups, showed differences in drug-induced gene expression connectivity scores. This could result in new therapeutic decision criteria based on the cluster or NLR ratio group to which each patient belongs. However, longitudinal pharmacodynamic analyses are required to verify the possible differential effects and the actual efficacy of the drugs *in vivo* or *in vitro* for each of the subgroups.

With few exceptions, the connectivity scores obtained for the drugs commonly used in SLE did not reach significant values. This could be a reflection of the low efficacy in practice that these drugs have on patients, since they target very general inflammatory processes^19^, regardless of the mechanisms specifically altered in each patient. Our approach allowed us to obtain not only drugs directed to the biological pathways altered during the disease, but, taking into account the heterogeneity of the gene signatures that the patients have, also allowed us to make the groups more molecularly and functionally homogeneous and to assign to a given group better drug candidates. Based on the heterogeneity of the SLE directed by neutrophils or lymphocytes, we should ideally consider two concepts when choosing a certain drug for a patient. Depending on the subgroup of SLE to which a patient belongs, we could look for drugs that reverse the altered gene patterns in that specific subgroup, but also, we could ascertain drugs whose targets are more specific or expressed in the characteristic cell type of the subgroup, unless their expression is ubiquitous or non-specific. Following these criteria, it could be of interest to test drugs such as kenpaullone or reversine, if available and if non-toxic, whose targets are expressed in neutrophils and lymphocytes alike and which appear to reverse the genetic patterns of all subgroups of SLE. Similarly, knowing the subgroup to which the patient belongs, for the lymphocyte-driven subgroup, inhibitors of mTOR and PI3K, whose targets in turn are expressed to a greater degree in lymphocytes, such as AZD-8055, WYE-354 or KU-0063794, could be used. Recent studies have shown the relationship between the over-expression of the mTOR pathway with not only a greater proliferation and survival of lymphocytes, but also with their hyperactivity in SLE^20^. The best candidates for the neutrophil-driven groups act on different pathways of inflammation. For example, the RHO-kinase inhibitor III, which in addition to its targets being more highly expressed in neutrophils, acts on macroautophagy, with important implications on its use as therapy and for which there is mounting evidence showing their efficacy^18,21,22^.

The drugs used on patients are homogeneously distributed among neutrophil and lymphocyte-related groups, that is, no enrichment was observed^4^. Similarly, we cannot determine using clinical data if any of these drugs is more effective in one group than in another because we do not have an objective parameter of patient improvement, as well as many sources of variation, such as the condition of patient in which the drug is applied (SLEDAI) or the different doses used. In addition, we find very few time points where a single drug is used, but they are found in combination with others. This is why more specific experimental studies are required to measure whether a certain drug is actually more effective within a subgroup or another.

Therefore, we propose obtaining the best candidate drugs based on their ability to reverse a patient’s gene signature depending on their assignment to the groups. This requires incorporation of the information into which subset each patient belongs as part of the criteria for a therapeutic decision. Although this work is an exploratory analysis and specific tests and clinical trials would be necessary to determine the utility of the classification from the clinical perspective, particularly in matters of non-response, primarily to test if the classification improves the actual therapies patients undergo.

## Methods

### SLE datasets

The analysis based on SLE transcriptomic profiles was carried out with the two independent SLE sets described previously^4^. The first, named Cohort1 is an adult set of patients from the SPARE^23^ study protocol approved by Johns Hopkins University School of Medicine Institutional Review Board. Patients were enrolled from the Hopkins Lupus Cohort after informed consent was obtained. The second dataset, or Cohort2, was collected from NCBI GEO GSE65391^24^ and has longitudinal gene expression data from pediatric human samples. The selection of gene expression profiles was done as described (1).

To study treatment differences in active lupus, we kept only the visit with the highest SLEDAI score (or highest SELENA-SLEDAI for Cohort1) for each patient, as long as this score was greater than 5, taking this value to indicate (relatively) high disease activity^25^. Finally, we only conserved the patients used in the stratification study^4^, in order to establish a direct relationship between drug repurposing and the clusters.

For the classification model based exclusively on clinical information, we included three new longitudinal cohorts. The GLADEL multinational Latin American prospective inception cohort^26^ (called Cohort3), that collected clinical data from a total of 1480 patients, 150 patients from Instituto Nacional de Ciencias Médicas y Nutrición of Mexico (Cohort4), and 30 patients from the Division of Medicine, University College London (Cohort5). The 5 cohorts including the pediatric and the Johns Hopkins, totaled over 2120 patients with differential biometry information for both, lymphocytes and granulocytes, presence or absence of nephritis, and the SLEDAI disease activity index measured at different time points (a minimum of 3) for each patient. Supplementary Dataset, Sheet 1, contains a table that summarizes the information of the cohorts.

All research was performed in accordance with standard clinical practice. Patient recruitment has ethical approval for each of the cohorts. All patients included in the study have given their informed consent.

### Clustering of SLE patients based on drug connectivity scores

The first step was to check whether, based on the connectivity scores between drug and patient signatures, the patients stratified in a similar fashion to the longitudinal stratification described previously^4^.

Gene expression fold changes were estimated for each patient from Cohort1 and Cohort2 with respect to the mean expression of healthy controls and the top 150 more over- and under-expressed genes were selected as a disease signature for each patient. Then, each signature was queried in CLUE to obtain the connectivity or similarity scores between patients and each drug-induced profile. The absolute rank sum^4^ was applied to select the drugs that had significantly high similarity score in absolute value across most of the patients and build a drug by patient connectivity score matrix from each cohort. Finally, a consensus clustering algorithm was implemented on ConsensusClusterPlus R package^27^ was applied to each matrix to establish groups of patients that shared similar drug connectivity scores profiles.

### Drug-repurposing analysis in SLE subgroups

From the cluster assignation previously described for Cohort 1 and 2 in our previous work^4^ we defined the set of differentially expressed genes in each SLE subset with respect to control samples using linear fixed models^28^. We then defined the common signature for the same group in Cohort 1 and 2 selecting maximum p value combinations from the two cohorts^29^ and the the top 150 significant over and under- expressed genes were used as the input signature in CLUE. Drugs with connectivity scores higher than 90 in absolute value (> = |90|) were selected for further analysis.

### Analysis of common mecahinsms of actions in SLE subgroups and drug target gene expression

**Figure 5:**
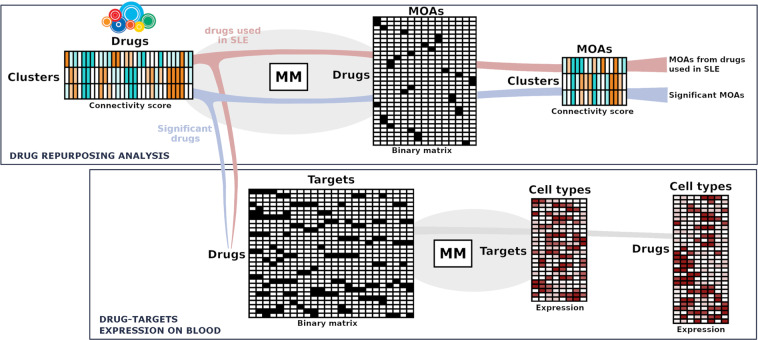
Summary and flow of the drug connectivity analyses. Based on the connectivity scores obtained in CLUE for each drug within each cluster, we analyzed on the one hand the drugs commonly used in SLE (red branch) and on the other hand, the drugs with score > = 90 in absolute value (blue branch), from which we extracted the significant MOAs by means of multiplication of matrices (MM). Subsequently, by matrix multiplication we obtained the relative expression of the targets of each drug in the blood cell types. The pairs of matrices that are multiplied are linked in the diagram by a shaded circle.

We used a matrix multiplication approach between the cluster by drugs connectivity matrix and a binary drug by mechanism of action (MOAs), binary matrix from information contained in CLUE database. This yielded a cluster by MOA matrix that was used to analyze the main MOAs of drugs associated with each SLE goup (selecting those with coefficients higher than 90). This framework was also used to analyze gene expression patterns in blood cell populations of drug targets. To this end, we multiplied a drug by target binary matrix with information from CLUE and a target by cell type gene expression matrix generated mining programmatically the Expression Atlas Database^30^ using a custom python script. A summary of all these processes is shown in Fig. 5.

Finally, we analyzed the similarities between MOAs using the Touchstone tool from CLUE. This tool ranks all drugs stored in CLUE based on the similarity score with an individual drug. Each significant drug was queried on Touchstone. We substituted the ranked drugs for their MOAs and applied a Gene Set Enrichment Analysis (GSEA)^31^ to obtain what were significantly similar MoAs (p value <  = 0.05) and different from the drug in question, that is, MOAs with positive and negative scores from GSEA, respectively.

With these analyses the connectivity scores of the drugs was obtained for each of the subgroups, the significant MOAs and the similarities between them, as well as the specificity of each drug in the blood cell types.

### Functional analysis

We analyzed the gene signatures obtained from SLE subgroups (top 150 most over and under-expressed genes) using Enrichr web tool^32^ and GENECODIS (http://genecodis.genyo.es/)^33^ to obtain lists of gene ontology biological processes that were over and under-represented for patients from each longitudinal cluster. ReviGO^34^ was used to cluster and visualize the ontological terms based on semantic similarity and to reduce term redundancies.

### Machine learning classifier based on clinical data

We used clinical variables from Cohort1 to train and generate a logistic regression-based model to classify new longitudinal patients (at least with data for 3 time points) within neutrophil or lymphocyte-driven SLE subgroups. On the training set we applied k-fold cross-validation with 1000 iterations using caret R package^35^, testing models with different variable combinations in order to search the model more accurately. Considering that the clusters we found were importantly related to neutrophil and lymphocyte counts and/or percentages, we used as variables the neutrophil and lymphocyte correlation with SLEDAI and the correlation between NLR and SLEDAI^36,37^. We also altered the value of k (i.e. percentage of patients that are used to internally test the model) to obtain the stability of the model under changes on sample size. Then, we tested the model on Cohort2 to obtain their sensitivity and specificity. Finally, we tested the model to classify patients from Cohort 3, 4 and 5 into neutrophil or lymphocyte-driven SLE.

Using the patient classification, the odds-ratios (OR) of patients with and without nephritis was calculated comparing subgroups in each cohort applying a random effect meta-analysis^38^ model to obtain the combined OR across cohorts.

## Supplementary information

Supplementary Information

Supplementary Dataset
