# Supplementary material for: Differential Treatments Based on Drug-induced Gene Expression Signatures and Longitudinal Systemic Lupus Erythematosus Stratification
Source: Sci Rep. 2019 Oct 29;9:15502. doi: 10.1038/s41598-019-51616-9 (PMC6820741; doi:10.1038/s41598-019-51616-9)
Supplement: Supplementary file 1 — Supplementary Information [file 41598_2019_51616_MOESM1_ESM.pdf]

Differential Treatments Based on Drug-induced Gene Expression Signatures and Longitudinal Systemic Lupus Erythematosus Stratification

Daniel Toro-Domínguez, Raúl Lopez-Domínguez, Adrián García Moreno, Juan A. Villatoro-García, Jordi Martorell-Marugán, Daniel Goldman, Michelle Petri, Daniel Wojdyla, Bernardo A. Pons-Estel, David Isenberg, Gabriela Morales-Montes de Oca, María Isabel Trejo-Zambrano, Benjamín García González, Florencia Rosetti, Diana Gómez-Martín, Juanita Romero-Díaz, Pedro Carmona-Sáez and Marta E. Alarcón-Riquelme.

Supplementary Information

Supplementary Figure 1: Consensus cluster results.

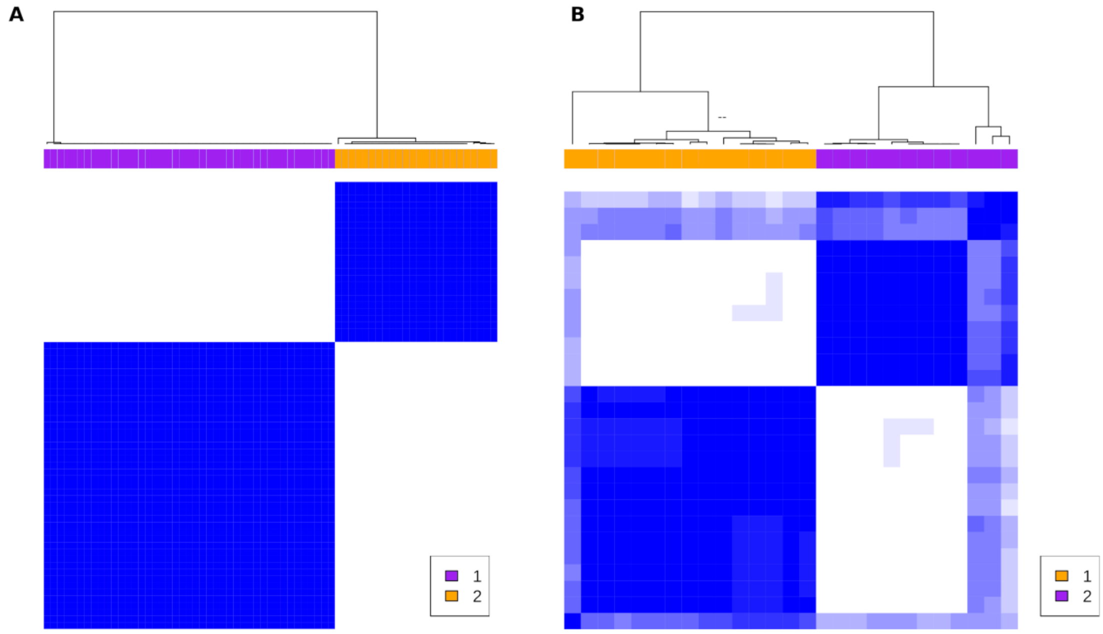

Results from consensus cluster for cohort1 (A) and cohort2 (B). The Bayesian information criterion was used to evaluate the optimal number of clusters. Patients are in columns and rows in the same order. Blue intensity represents the probability of two patients clustering together.

Supplementary Figure 2: Functional analysis from Revigo.

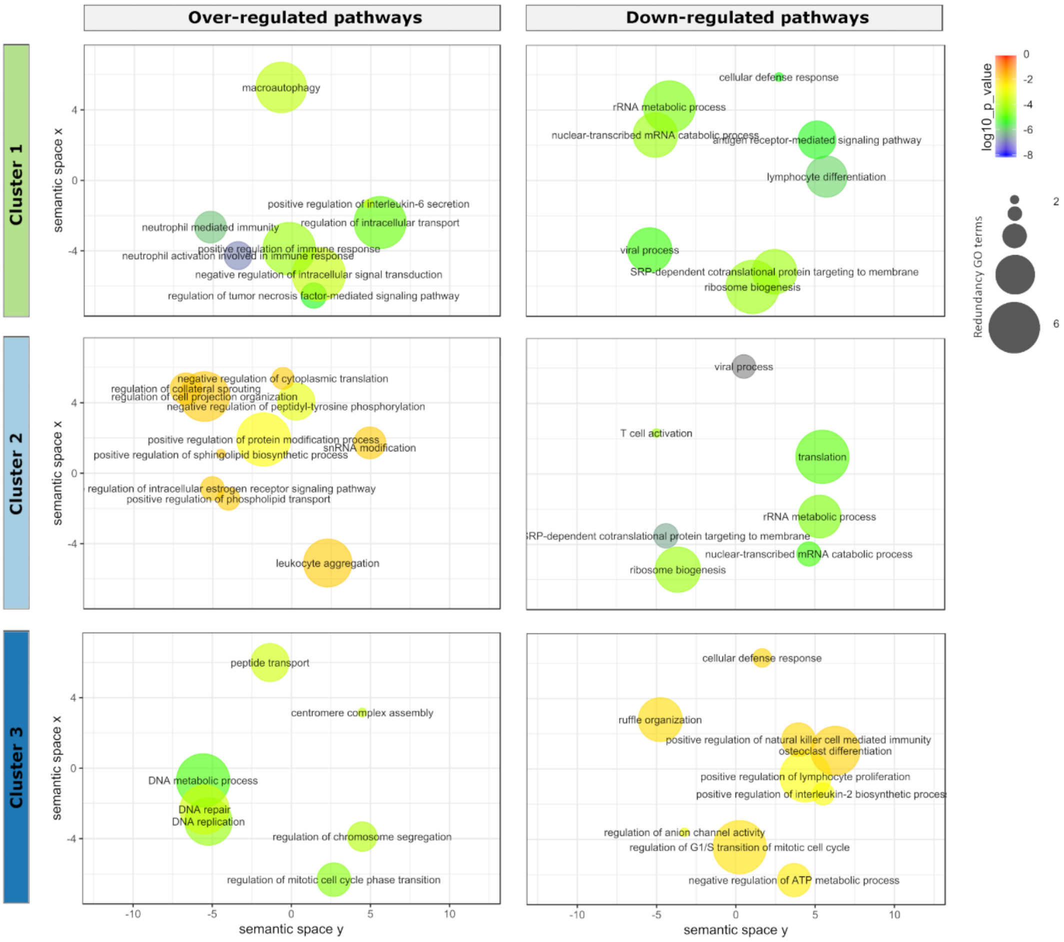

The functional pathways significantly over-expressed (left) and under-expressed (right) for each cluster semantically clustered using the Revigo webtool. The color scale reflects the significance level and the size of the circles represents the redundancy explained by each clustered term. Supplementary File 1, sheets 2-4 contains all significant pathways.

Supplementary Figure 3: Internal model testing.

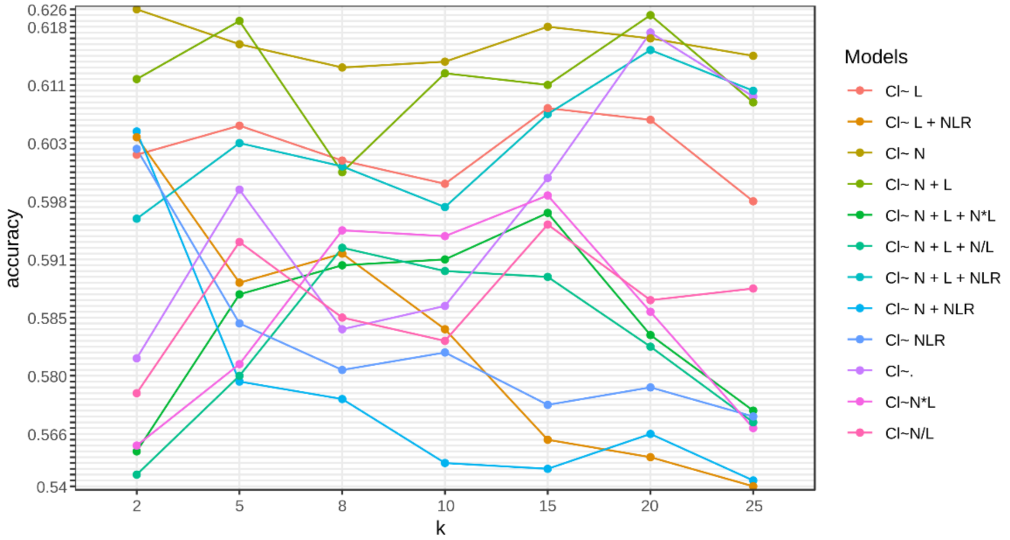

*D*ifferent variable combinations used on the classification model and the accuracy obtained (y-axis) across different k values (x-axis). Cl: cluster label. N: correlation between neutrophils and SLEDAI. L: correlation between lymphocytes and SLEDAI. NLR: correlation between neutrophil/lymphocyte ratio and SLEDAI.

Supplementary dataset: Cohorts and functional analysis of the results.

Excel file that contains 4 sheets. Sheet 1 shows a table with a description of SLE cohorts and their use in the analyses. Sheets 2-4 contain the significant under and over-expressed gene ontology terms in the 3 SLE clusters.
